# Tropomodulin1 Expression Increases Upon Maturation in Dendritic Cells and Promotes Their Maturation and Immune Functions

**DOI:** 10.3389/fimmu.2020.587441

**Published:** 2021-01-15

**Authors:** Xianmei Liu, Xue Xia, Xifu Wang, Jing Zhou, Lanping Amy Sung, Jinhua Long, Xueyu Geng, Zhu Zeng, Weijuan Yao

**Affiliations:** ^1^School of Basic Medical Sciences, School of Biology and Engineering, Guizhou Medical University, Guiyang, China; ^2^Hemorheology Center, Department of Physiology and Pathophysiology, School of Basic Medical Sciences, Peking University Health Science Center, Beijing, China; ^3^Department of Emergency, Beijing Anzhen Hospital, Capital Medical University, Beijing, China; ^4^Department of Bioengineering, University of California, San Diego, La Jolla, CA, United States; ^5^Department of Integration of Chinese and Western Medicine, School of Basic Medical Science, Peking University Health Center, Beijing, China

**Keywords:** dendritic cells, maturation, Tmod1, TLR4 signaling, antigen presentation

## Abstract

Dendritic cells (DCs) are the most potent antigen-presenting cells. Upon maturation, DCs express costimulatory molecules and migrate to the lymph nodes to present antigens to T cells. The actin cytoskeleton plays key roles in multiple aspects of DC functions. However, little is known about the mechanisms and identities of actin-binding proteins that control DC maturation and maturation-associated functional changes. Tropomodulin1 (Tmod1), an actin-capping protein, controls actin depolymerization and nucleation. We found that Tmod1 was expressed in bone marrow-derived immature DCs and was significantly upregulated upon lipopolysaccharide (LPS)-induced DC maturation. By characterizing LPS-induced mature DCs (mDCs) from Tmod1 knockout mice, we found that compared with *Tmod1^+/+^* mDCs, Tmod1-deficient mDCs exhibited lower surface expression of costimulatory molecules and chemokine receptors and reduced secretion of inflammatory cytokines, suggesting that Tmod1 deficiency retarded DC maturation. Tmod1-deficient mDCs also showed impaired random and chemotactic migration, deteriorated T-cell stimulatory ability, and reduced F-actin content and cell stiffness. Furthermore, Tmod1-deficient mDCs secreted high levels of IFN-β and IL-10 and induced immune tolerance in an experimental autoimmune encephalomyelitis (EAE) mouse model. Mechanistically, Tmod1 deficiency affected TLR4 signaling transduction, resulting in the decreased activity of MyD88-dependent NFκB and MAPK pathways but the increased activity of the TRIF/IRF3 pathway. Rescue with exogenous Tmod1 reversed the effect of Tmod1 deficiency on TLR4 signaling. Therefore, Tmod1 is critical in regulating DC maturation and immune functions by regulating TLR4 signaling and the actin cytoskeleton. Tmod1 may be a potential target for modulating DC functions, a strategy that would be beneficial for immunotherapy for several diseases.

## Introduction

Dendritic cells (DCs) are the most potent antigen-presenting cells (APCs) and play critical roles in initiating and exaggerating innate and adaptive immune responses (1, 2). DC-based immunotherapy against cancer and autoimmune diseases has achieved promising clinical outcomes (3–6). Originating from bone marrow precursors, immature DCs (imDCs) reside in the peripheral non-lymphoid tissues and execute the immune surveillance function (7). Upon capturing antigens through pattern recognition receptors such as Toll-like receptors (TLRs), imDCs gradually develop into mature DCs (mDCs) through the activation of MyD88- and TRIF-dependent signaling pathways (8, 9). This occurs in concert with the translocation of MHC-II from endosomal compartments to the plasma membrane, and the upregulation of costimulatory molecules, chemokine receptors, and cytokines (10–13). Simultaneously, mDCs migrate to the lymphoid organs and present the antigenic peptides to naïve T cells to mediate immune responses (14, 15). Although a considerable amount of information about the biological behaviors of DCs is available, the molecular mechanisms underlying their differentiation, maturation, and immune functions of DCs remain elusive.

The Actin cytoskeleton undergoes remodeling during DC maturation, and plays critical roles in regulating the morphology, adhesion, migration, endocytosis, and antigen presentation (16–19). As regulators of the actin cytoskeleton, many actin-binding proteins are found differentially expressed during DC maturation and contribute to the maturation-associated functional changes. For example, the actin bundling protein, Fascin1, is upregulated upon maturation and promotes the migration of mDCs (20). The activation of Cofilin is correlated with the morphological changes during DC maturation (21). Members of the Rho family GTPases, Rac1 and Rac2, control the formation of dendrites and their T-cell priming in mDCs (22). Another Rho family GTPase, Cdc42, stops expressing during DC maturation and regulates the secretory activity of mDCs (23). The scaffolding protein, Caveolin-1, is upregulated in DCs upon maturation and promotes their migration to lymph nodes through Rac1-dependent actin cytoskeleton remodeling to elicit effective T-cell responses (24). Interestingly, knockout of Fascin1 or Caveolin-1 does not alter the expression of DC maturation markers (20, 24). However, Cdc42 knockout results in phenotypically mature DCs, that express high levels of CD86 on the cell surface (23). These findings indicate that actin-binding proteins may play different roles in regulating the maturation process and immune functions of DCs, which needs further studies.

Tropomodulin1 (Tmod1) is an actin-capping protein that was first isolated in human erythrocytes (25). By binding to tropomyosin (TM) and actin, Tmod1 caps the pointed end of TM-coated actin filaments and decreases the rate of actin depolymerization (26). Tmod1 also has actin nucleating activity depending on its actin monomer binding and pointed end-capping activity (27, 28). Therefore, Tmod1 plays important roles in regulating actin dynamics and cytoskeleton structure, and further determines cell morphology, cell mechanics, contraction, and dendritic protrusion growing from neurons (29, 30). Knockout of Tmod1 in mice results in embryonic lethality because of non-contractile heart tube with disorganized myofibrils, accumulation of mechanically weakened primitive erythroid cells in the yolk sac, and failure of primary capillary plexus remodeling (31, 32). X-gal staining of *Tmod1^+/-^* mice with *lacZ* gene knock-in revealed that Tmod1 was expressed in mouse peripheral blood mononuclear cells (PBMCs) (33). Using microarray and RNA-sequencing techniques, several studies have revealed that Tmod1 is significantly upregulated in PBMCs or macrophages of patients with advanced renal cell carcinoma, inflammatory bowel diseases, or rheumatoid arthritis (34–36). These results indicate that Tmod1 is expressed in immune cells and is possibly involved in regulating inflammation and immune responses.

In the present study, we demonstrated that Tmod1 is expressed in bone marrow-derived dendritic cells (BMDCs) and is upregulated upon lipopolysaccharide (LPS)-induced DC maturation. Using BMDCs from *TOT/Tmod1^-/-^* mice, that were obtained by crossing cardiomyocyte-specific Tmod1 overexpressing transgenic (TOT) mice with *Tmod1^+/-^* mice (37, 38), we showed that Tmod1 deficiency retarded DC maturation by downregulating the expression of costimulatory molecules and inflammatory cytokines, and impaired their migration and T-cell stimulatory abilities by altering their actin cytoskeleton and cell mechanics. Furthermore, LPS-treated Tmod1-deficient DCs secreted high levels of IFN-β and IL-10, and induced immune tolerance in an experimental autoimmune encephalomyelitis (EAE) mouse model. Mechanistically, Tmod1 deficiency affected TLR4 signaling transduction, resulting in decreased activity of NFκB and MAPK pathways but increased activity of the IRF3 pathway. Rescue with exogenous Tmod1 reversed the effect of Tmod1 deficiency on TLR4 signaling. Therefore, Tmod1 is critical in regulating DC maturation and immune functions, possibly by regulating TLR4 signaling and the actin cytoskeleton.

## Materials and Methods

### Reagents and Antibodies

Recombinant murine granulocyte-macrophage colony stimulating factor (rmGM-CSF) and recombinant murine interleukin 4 (rmIL-4) were purchased from Peprotech (Rocky Hill, NJ, USA). LPS was purchased from Sigma (St. Louis, MO, USA). β-mercaptoethanol was obtained from MP Biomedicals LLC (Solon, OH, USA). Pam3csk4, poly (I:C), and CpG ODN1826 were purchased from InvivoGen (San Diego, CA, USA). FITC-, APC-, and PE-conjugated anti-murine CD4, CD11c, CD40, CCR7, CD80, CD86, and MHC-II antibodies were purchased from BioLegend (San Diego, CA, USA) and eBiosciences (New York, NY, USA). Antibodies against CD11c, TLR4, MyD88, and IκB were purchased from Biosynthesis Biotechnology Co. Ltd. (Beijing, China). Antibodies against p65 and p38 were obtained from Zhongshan Golden Bridge Co. Ltd. (Beijing, China). Antibodies against phospho-p65, phospho-IRF3, phospho-ERK1/2, and IRF3 were from Cell Signaling Technology (Danvers, MA, USA). Antibodies against phospho-p38 and ERK1/2 were purchased from Bioworld Technology (Nanjing, China). Anti-Tmod1 antibody was prepared by AbMax Biotechnology Co., Ltd. (Beijing, China). Antibodies against GAPDH and β-actin were obtained from Santa Cruz Biotech. (Santa Cruz, CA, USA). Tmod1 adenovirus and control adenovirus were constructed by SinoGeneMax Co. Ltd. (Beijing, China).

### Animals and Culture of BMDCs

A *Tmod1* knockout mouse model was first created by disrupting exon 1 in Dr. L. Amy Sung’s laboratory at the University of California, San Diego (31). While *Tmod1^-/-^* mice died at embryonic day 9.5, the heterozygous *Tmod1^+/-^* mice were able to mate with a cardiac specific Tmod1 overexpressing transgenic (TOT) mice (37). Interbreeding between their offsprings resulted in *TOT/Tmod1^-/-^* mice where the overexpression of *Tmod1* in the heart rescued the homozygosity’s lethality (38). *TOT/Tmod1^-/-^* mice were transferred from Dr. L. Amy Sung’s lab to the Peking University Health Science Center and maintained in SPF animal room. C57BL/6J (*Tmod1^+/+^*) mice were purchased from the animal department of the Peking University Health Science Center. The animal study was approved by the ethics committee of the Peking University Health Science Center. BMDCs were obtained from 8 week-old female *TOT/Tmod1^-/-^* and *Tmod1^+/+^* mice according to the method developed by Roney (39). Briefly, bone marrow cells were flushed out from freshly dissected femurs and tibias. Following lysis of the red blood cells in lysis buffer, the remaining cells were resuspended in Roswell Park Memorial Institute 1640 (RPMI 1640) medium supplemented with 10% fetal bovine serum, 1% penicillin-streptomycin, and 0.1% β-mercaptoethanol, and differentiated into imDCs in the presence of 20 ng/ml rmGM-CSF and 10 ng/ml rmIL-4 for 7 days. The imDCs were then matured into mDCs by adding 100 ng/ml LPS for another 2 days. The imDCs were also treated with Pam3csk4 (100 ng/ml), poly (I:C) (25 μg/ml), and CpG ODN 1826 (1 μM), respectively, for 1–2 days.

### Flow Cytometry

ImDCs and mDCs were fixed with 4% paraformaldehyde and labeled with FITC-, PE-, or APC-conjugated antibodies to mouse CD11c, CD86, MHC-II, CD80, CD40, and CCR7, and the corresponding isotype controls. The cells were then analyzed with a BD FACS Calibur™ flow cytometer (BD Biosciences, San Diego, CA, USA).

### Western Blotting

ImDCs and mDCs were harvested and lysed in RIPA buffer containing protease inhibitor cocktail and 1 mM phenylmethylsulfonyl fluoride (PMSF). Total proteins (100 μg) were separated on a 10% sodium dodecyl sulfate-polyacrylamide gel electrophoresis (SDS-PAGE) gel and transferred onto a nitrocellulose membrane. After blocking with 5% nonfat milk, the membrane was incubated with antibodies against Tmod1, GAPDH, CD11c, p65, p-p65, p38, p-p38, ERK1/2, p-ERK1/2, and TLR4 overnight at 4°C. After washing, the membranes were incubated with HRP-conjugated secondary antibody for 1 h at room temperature (25°C). The signals were visualized using an enhanced chemiluminescence (ECL) detection kit (Evergreen, Beijing, China) and exposed to X-ray films.

### Real-Time Quantitative PCR

Total RNA was extracted from imDCs and mDCs using RNAtrip reagent (Applygen Biotechnologies Inc., Beijing, China) and then reverse transcribed into cDNA using the First Strand cDNA Synthesis Kit (Thermo Fisher Scientific, Waltham, MA, USA). Real-time quantitative PCR (RT-qPCR) was performed on a Mx3000 Multiplex Quantitative PCR system (Stratagene, La Jolla, CA, USA) using EvaGreen qPCR Master Mix-low Rox (Applied Biological Materials (abm) Inc., Richmond, BC, Canada). The sequences of the primers are listed in **Table 1**. GAPDH was used as an internal control. A relative fold change in the gene expression was calculated using the method of 2^-△△Ct^ method.

**Table 1:** The list of primer sequences.

Tmod1	5’-GACACAGCCTCACACAATGT-3’/ 5’-CTTGGTGGTCTGATCCTTCT-3’
CD80	5’-GGCAAGGCAGCAATACCTTA-3’/ 5’-CCATGTCCAAGGCTCATTCT-3’
CD86	5’-GGCAGATATGCAGTCCCATT-3’/ 5’-AGAACTTACGGAAGCACCCA-3’
CD40	5’-CTGGCACAAATCACAGCACT-3’/ 5’-CTGCATGGTGTCTTTGCCT-3’
CCR7	5’- GAAGGCTGTGCTTTTGGTTC-3’/ 5’-CAAACAGGAGCTGATGTCCA-3’
MHC-II	5’-ACCGTGTTCTGCTCATCCT-3’/ 5’-AGTTCTCAAAGTAGTGCCT-3’
IL-6	5’-GATGGATGCTACCAAACTGGA-3’/ 5’-TCTGAAGGACTCTGGCTTTG-3’
TNF-α	5’- CAGCCTCTTCTCATTCCTGC-3’/ 5’-GGTCTGGGCCATAGAACTGA-3’
IL-12a	5’- CTAGACAAGGGCATGCTGGT-3’/ 5’-GCTTCTCCCACAGGAGGTTT-3’
IFN-γ	5’-ATGAACGCTACACACTGCATC-3’/ 5’-CCATCCTTTTGCCAGTTCCTC-3’
IFN-β	5’- AATTTCTCCAGCACTGGGTG-3’ 5’-AGTTGAGGACATCTCCCACG-3’
IL-10	5’- TACACCTGCGTTTCTCAGCC-3’/ 5’-CAGTATTGCACTCTGTAAGCCC-3’
TLR4	5’-TGTTCTTCTCCTGCCTGACA-3’/5’-TGTCATCAGGGACTTTGCTG-3’
Tmod2	5’-TTGGAGAGGGTGAAAGAGAGGG-3’/ 5’-GTGTTGGTAATGGGATTGGGAT-3’
Tmod3	5’-ACTATATCCCTCGACCCAGA-3’/ 5’-GACACCGTTACTACTTCCCA-3’
Tmod4	5’-GTGATGCGGTAGAGATGGAGAT-3’/ 5’-CTTGTTGGTAAAAGGAAGGGTG-3’
GAPDH	5’- ACCACAGTCCATGCCATCAC-3’/ 5’-TCCACCACCCTGTTGCTGTA-3’

### Adenovirus Infection on Tmod1-Deficient DCs

About 5 × 10^5^ *TOT/Tmod1^-/-^* imDCs were infected with Tmod1 adenovirus (Ad-Tmod1) or control adenovirus (Ad-Null) at 200 MOI (multiplicity of infection) for 6 h. Equal number of *TOT/Tmod1^-/-^* imDCs without adenovirus treatment were used as the negative control. The cells were further treated with LPS (100 ng/mL) for 24 h before cells and culture media were collected.

### Measurement of Cyotkines and Chemokines by Multiplex Analysis or Enzyme Linked Immunosorbent Assay

Culture media of imDCs, mDCs, and OVA peptide-treated spleen cells were collected. The levels of cytokines and chemokines, including interleukin-6 (IL-6), tumor necrosis factor-α (TNF-α), interleukin-12 (IL-12), interferon-γ (IFN-γ), interferon-β (IFN-β), and interleukin-10 (IL-10), were measured by flow cytometry using LEGENDplex™ bead-based immunoassay kits (BioLegend). The levels of TNF-α and IL-10 in the culture media of adenovirus-infected and LPS-treated *TOT/Tmod1^-/-^* imDCs and TLR agonist-treated imDCs were measured using *Enzyme linked immunosorbent assay* (ELISA) kits (BOSTER Biotechnology, Wuhan, China).

### Endocytosis Assay for imDCs

Approximately 5 × 10^5^ imDCs were incubated with 500 μg/ml FITC-dextran particles (43.2 kDa, Sigma) in 1 ml of RPMI 1640 at 4°C or 37°C (40). After 0.5 or 2 h of incubation, the cells were washed three times with PBS to remove the un-endocytosed particles and the endocytosed particles were analyzed using flow cytometry. The differences in the fluorescence intensities between 37°C and 4°C represented the endocytosis abilities of imDCs.

### Migration Assay

Migration of imDCs and mDCs was measured using a modified Boyden chamber (5-μm pore size). A total of 2 × 10^5^ cells in 200μl volume was plated in the upper compartment of the chamber, and 600μl of complete medium supplemented with or without 200 ng CCL19 was added to the lower compartment. The chamber was incubated for 12 h at 37°C, following which the migrated cells were counted under a light microscope.

### Allogeneic Mixed Lymphocyte Reaction

Mixed lymphocyte reaction (MLR) assay was used to determine the T-cell activation abilities of the mDCs (40). Allogeneic T cells were obtained from the spleen cells of C57BL/6J mice by passing the spleen cells through a nylon wool column. mDCs (1 × 10^5^, 1 × 10^4^, and 1 × 10^3^) were mixed with 1 × 10^5^ T cells, respectively, in a total volume of 200μl, and the mixtures were incubated in a CO_2_ incubator for 48 h. Then, 20μl CCK8 solution (Enhanced Cell Counting Kit-8, Beyotime Biotech., Shanghai, China) was added to each mixture for 4 h. The proliferation of the T cells was quantified by measuring the optical density at 450 nm in a microplate reader (BioRad, Hercules, CA, USA).

### Adoptive Transfer With DCs and *In Vitro* Restimulation of T Cells

Mature DCs were incubated with 10 μg/ml OVA peptide (323–339) (Alpha Diagnostic Intl Inc., San Antonio, TX, USA) at 37°C for 2 h. The cells were washed twice and resuspended in serum-free RPMI 1640 medium. Treated mDCs (1 × 10^6^) were injected intravenously into C57BL/6J mice on days 1, 3, and 5. On day 8, the mice were sacrificed, and their spleen cells were collected, and re-stimulated with 10 μg/ml OVA peptide (323–339) at 37°C for 3 days (41). Then, the spleen cells were washed with PBS, stained with FITC-conjugated anti-mouse CD4 antibody, and further analyzed by flow cytometry. The fluorescence intensity indicated the proliferation of CD4^+^ T cells. The concentrations of IFN-γ and IL-10 in culture medium of OVA-peptide-treated spleen cells were measured by flow cytometry using LEGENDplex™ bead-based immunoassay kits (BioLegend).

### Preparation of Myelin Oligodendrocyte Glycoprotein (MOG)35-55 Peptide-Pulsed mDCs and Induction of EAE

Mature DCs were pulsed with 20 μg/ml MOG35-55 peptide (Sigma) for 1 h at 37°C (41). The pulsed cells (1.2 × 10^6^) were injected intravenously into female C57BL/6J mice twice (on days 0 and 4). Experimental autoimmune encephalomyelitis (EAE) was induced by subcutaneous injection of 100 μg of MOG35-55 peptide emulsified in complete Freund’s adjuvant (Chondrex, Redmond, WA, USA) with 100 μl of 4 mg/ml *Mycobacterium tuberculosis* (Chondrex) on days 7 and 14. In addition, 200 ng of pertussis toxin (Invitrogen, Carlsbad, CA, USA) was injected intraperitoneally on days 7 and 9. The mice were observed and scored on the scale of 0 to 5: 0, no symptoms; 1, flaccid tail; 2, hind limb weakness; 3, partial hind limb paralysis; 4, complete hind limb paralysis; 5, moribund state (41).

### Measurement of F-Actin Content in the DCs

ImDCs and mDCs were fixed in 4% paraformaldehyde and washed twice with PBS. After permeabilization with 0.1% Triton X-100, the cells were blocked with 1% bovine serum albumin (BSA) for 30 min at room temperature. The cells were then stained with 0.165 μM rhodamine phalloidin (Invitrogen) in the dark for 20 min. The cells were washed, resuspended in PBS, and analyzed using a BD FACS Calibur (BD Bioscience). The mean fluorescence intensity represented the F-actin content in the cells.

### Laser Scanning Confocal Microscopy

ImDCs and mDCs were cultured on poly L-lysine-treated coverslips and stained with rhodamine phalloidin as mentioned above. The nuclei were stained with 4’,6-diamidino-2-phenylindole (DAPI, Beyotime Biotech., Shanghai, China) for 5 min. After washing with PBS, the cells were observed under a laser scanning confocal microscope (Leica TCS SP8 MP FLIM, Wetzlar, Germany).

### Measurement of Young’s Moduli of the DCs by Nanoindenter

ImDCs and mDCs were cultured on poly-L-lysine-treated coverslips. The petri dish containing the coverslip was placed on an inverted microscope equipped with a nanoindenter (Piuma Chiaro, Optic II, Amsterdam, Netherlands). A probe with spring constant of 0.18 N/m and a spherical tip radius of 9-μm was mounted on the cantilever. During indentation, the tip was brought into contact with the cell surface and load-indentation and load-time data were recorded. The indentation depth was 10µm and the loading and unloading time was set to 2 s. The loading and unloading curves were fitted with Hertzian contact model and Young’s modulus was calculated. The mean Young’s modulus for each specimen was generated from at least 20 to 30 cells from three independent preparations.

### Statistical Analysis

All experimental procedures were repeated at least three times and are presented as mean ± standard error of mean (SEM). GraphPad Prism 7.0 software was used for the normal distribution test, homogeneity of variance test, statistical analysis, and plotting. Paired or unpaired Student’s *t*-tests were used to compare the results of the two groups, and analysis of variance was used between the groups. P < 0.05 was considered statistically significant.

## Results

### Tmod1 Is Expressed in BMDCs and Upregulated Upon LPS-Induced Maturation

**Figure 1:**
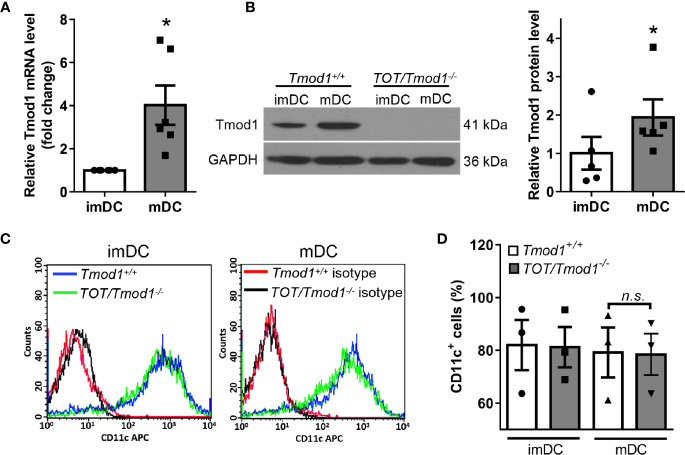
The expression of Tmod1 increased upon maturation in dendritic cells (DCs) induced by lipopolysaccharide (LPS) and Tmod1 deficiency did not influence DC differentiation. **(A)** Bone marrow-derived immature DCs (imDCs) of wild type (*Tmod1^+/+^*) mice were treated with LPS (100 ng/ml) for 2 days to induce the maturation. The mRNA level of Tmod1 in imDCs and mature DCs (mDCs) was detected by qPCR. **(B)** ImDCs from *Tmod1^+/+^* and TOT/Tmod1^-/-^ mice were induced to mature DCs (mDCs) with LPS. The protein level of Tmod1 in imDCs and mDCs of both genotypes was detected by western blotting. Tmod1 expression in *Tmod1^+/+^* imDCs and mDCs was quantified after normalization to GAPDH (right panel). **(C)** The imDCs and mDCs from *Tmod1^+/+^* and *TOT/Tmod1^-/-^* mice were stained with APC-conjugated antibody for DC marker, CD11c, and analyzed with flow cytometry. The representative flow cytometry histograms were shown. **(D)** The percentages of CD11c^+^ cells were obtained from flow cytometry data and plotted. Data are presented as mean ± SEM. *p < 0.05, *n.s.*, no statistic significance; paired, two-tailed student’s *t*-test in **(A, B)**, un-paired, two-tailed student’s *t*-test in **(C)**.

To define the immune functions of Tmod1, we first examined whether Tmod1 was expressed in DCs. We isolated bone marrow cells from C57BL/6J (*Tmod1^+/+^*) mice and differentiated them into imDCs following treatment with IL-4 and GM-CSF. LPS was used to induce DC maturation. qPCR data showed that compared with imDCs, Tmod1 was upregulated by approximately 4-fold in mDCs (**Figure 1A**, p < 0.05). Western blotting data further confirmed that Tmod1 was present in imDCs and its expression level increased significantly upon DC maturation (**Figure 1B**, p < 0.05). We wondered whether agonists for other TLRs could also promote Tmod1 expression. Therefore, imDCs of *Tmod1^+/+^* mice were treated with TLR2 agonist Pam3csk4 and TLR3 agonist poly (I:C). Un-treated and LPS-treated imDCs served as a negative and positive control. Both qPCR and western blotting data showed that neither Pam3csk4 nor poly (I:C) could stimulate Tmod1 expression (**Figures S1A, B**). This suggests that LPS is the strongest stimulation for Tmod1 expression among these TLR agonists.

We also cultured DCs of *TOT/Tmod1^-/-^* mice and analyzed Tmod1 expression in these cells. However, no Tmod1 could be detected, suggesting the complete absence of Tmod1 in *TOT/Tmod1^-/-^* DCs (**Figure 1B**). In addition to Tmod1, three homologs of similar size exist in Tmod family, Tmod2 (neuronal specific), Tmod3 (ubiquitous), and Tmod4 (skeletal muscle specific) (42). Compensatory effect is observed among some of the homologs (43). To examine the expression of other Tmod homologs in Tmod1-deficient DCs, we performed qPCR on imDCs and LPS-induced mDCs of *Tmod1^+/+^* and *TOT/Tmod1^-/-^* mice. Results showed that the expression levels of *Tmod2*, *Tmod3*, and *Tmod4* were all comparable between the two genotypes (**Figure S2**). This suggests that other Tmod homologs did not compensate for the loss of Tmod1.

### Tmod1 Is Not Essential for the Differentiation of BMDCs

Since Tmod1 is expressed in both imDCs and mDCs, we sought to determine whether Tmod1 plays a role in DC differentiation. We measured the expression level of the DC marker, CD11c, on the surface of imDCs and mDCs from *Tmod1^+/+^* and *TOT/Tmod1^-/-^* mice. Flow cytometry data showed that CD11c levels were comparable in *Tmod1^+/+^* and *TOT/Tmod1^-/-^* DCs, regardless of their state of maturation (immature or mature; **Figures 1C, D**). We also analyzed CD11c levels in the cell lysates of *Tmod1^+/+^* and *TOT/Tmod1^-/-^* DCs using western blotting, but did not detect any difference (**Figure S3**). These results suggest that Tmod1 is not essential for the differentiation of BMDCs.

### Tmod1 Is Required for LPS-Induced Maturation and Cytokine Secretion in DCs

**Figure 2:**
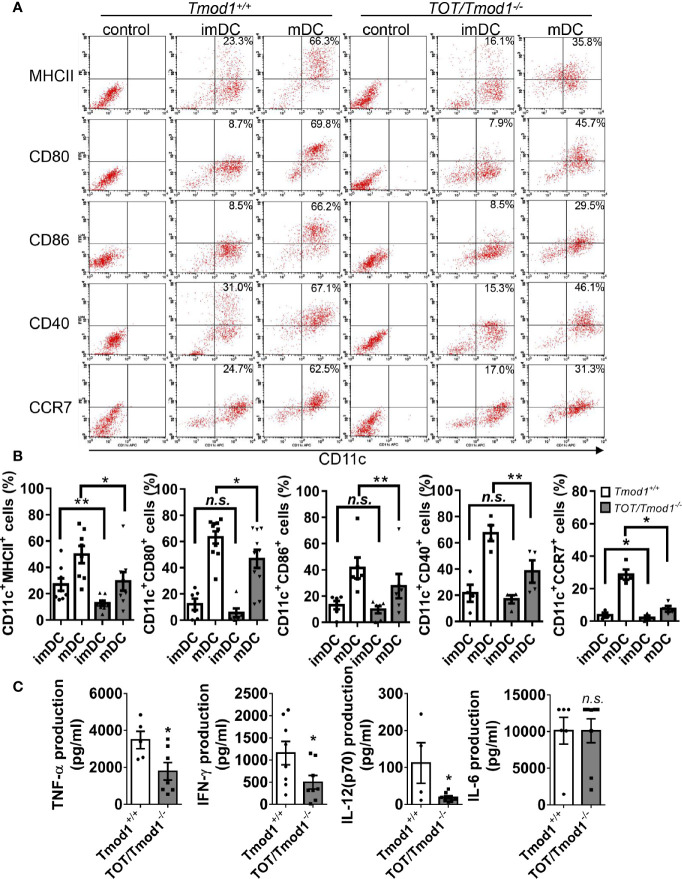
Tmod1 is required for lipopolysaccharide (LPS)-induced costimulatory molecules and chemokine receptor expression and cytokine secretion in dendritic cells (DCs). **(A)** The immature DCs (imDCs) from *Tmod1^+/+^* and *TOT/Tmod1^-/-^* mice were treated with 100 ng/ml LPS for 2 days, respectively, to induce maturation. Both imDCs and mature DCs (mDCs) of two genotypes were double-stained with APC-conjugated CD11c antibody and FITC- or PE-conjugated antibodies for MHC-II, CD80, CD86, CD40, and CCR7, and then analyzed with flow cytometry. imDCs of both genotypes were stained with isotype controls and run on flow cytometry to determine the gating. Representative dot plots were shown and the percentages of double-positive cells were labeled. **(B)** The statistical results for the percentages of CD11c^+^/MHC-II^+^, CD11c^+^/CD80^+^, CD11c^+^/CD86^+^, CD11c^+^/CD40^+^, and CD11c^+^/CCR7^+^ cells for imDCs and mDCs from *Tmod1^+/+^* and *TOT/Tmod1^-/-^* mice were shown. **(C)** ImDCs from *Tmod1^+/+^* and *TOT/Tmod1^-/-^* mice were induced to mature by treatment with 100 ng/ml LPS for 2 days and then culture media were collected. The concentrations of cytokines, TNF-α, IFN-γ, IL-12(p70), and IL-6, in the culture media were measured on a flow cytometer by using a bead-based immunoassay kit. Data are presented as mean ± SEM. *p < 0.05, **p < 0.01, *n.s.*, no statistical significance; unpaired, two-tailed student’s *t*-test.

The finding that Tmod1 was upregulated upon maturation suggests that Tmod1 may be involved in the maturation process and the functional changes related to maturation. DC maturation is characterized by increased surface expression of costimulatory molecules and chemokine receptors as well as the secretion of cytokines. Therefore, we examined the expression of MHC-II, CD80, CD86, CD40, and CCR7 on Tmod1-deficient DCs using flow cytometry. The results showed that Tmod1-deficient imDCs had considerably lower expression of MHC-II and CCR7 than *Tmod1^+/+^* imDCs (**Figures 2A, B**). However, the expression of CD80, CD86, and CD40 was similar between the two groups. Following LPS induction, the expression of MHC-II, CD80, CD86, CD40, and CCR7 increased markedly on both *Tmod1^+/+^* and *TOT/Tmod1^-/-^* mDCs. However, the expression levels of these molecules were significantly lower on *TOT/Tmod1^-/-^* mDCs than on *Tmod1^+/+^* mDCs (**Figures 2A, B**), suggesting that *TOT/Tmod1^-/-^* mDCs were phenotypically less mature than *Tmod1^+/+^* mDCs. We also compared the levels of cytokines secreted by *Tmod1^+/+^* and *TOT/Tmod1^-/-^ m*DCs. The data showed that compared with *Tmod1^+/+^m*DCs, *TOT/Tmod1^-/-^ m*DCs secreted noticeably less TNF-α, IFN-γ, and IL-12 (p70) (p < 0.05, **Figure 2C**). IL-6 secretion was similar in both groups. These results indicate that Tmod1 deficiency retarded LPS-induced DC maturation by reducing surface marker expression and cytokine secretion.

### Tmod1 Regulates LPS-Induced Phosphorylation of NF-κB and p38 MAPK in DCs

**Figure 3:**
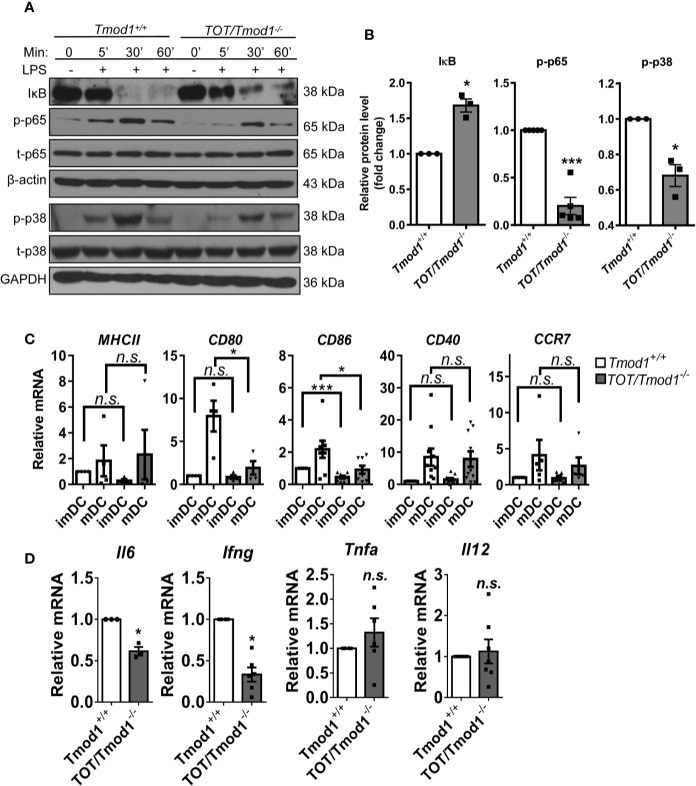
Tmod1 regulates lipopolysaccharide (LPS)-induced phosphorylation of NF-κB and p38 MAPK and the expression of downstream costimulatory molecules and cytokines in DCs. **(A)** The immature DCs (imDCs) from *Tmod1^+/+^* and *TOT/Tmod1^-/-^* mice were treated with LPS (100 ng/ml) for 0, 5, 30, 60 min, respectively, and then total proteins were extracted. The expression of IκB, phosphorylated p65 (p-p65), total p65 (t-p65), phosphorylated p38 (p-p38), and total p38 (t-p38) were detected by western blotting by using specific antibodies. β-actin and GAPDH were used as internal controls. **(B)** The expressions of IκB, p-p65, and p-p38 at 5 min after LPS treatment were quantified after normalization to β-actin, t-p65, or t-p38. Their relative protein levels were presented as fold change. **(C)** The imDCs from *Tmod1^+/+^* and *TOT/Tmod1^-/-^* mice were matured by LPS treatment for 2 days. Total RNAs were extracted from imDCs and mature DCs (mDCs) of both genotypes and qPCR was performed to detect the expression of *MHCII, CD80, CD86, CD40*, and *CCR7*. **(D)** The mRNA expression of cytokines, IL-6 (*Il6*), IFN-γ (*Ifng*), TNF-α (*Tnfa*), and IL-12 (*Il12*), in LPS-induced mDCs from *Tmod1^+/+^* and *TOT/Tmod1^-/-^* mice were detected by qPCR. Data are presented as mean ± SEM. *p < 0.05, ***p < 0.001, *n.s.*, no statistical significance; paired, two-tailed student’s *t*-test.

The decreased expression of multiple surface markers and secretion of several cytokines suggests that Tmod1 deficiency may affect the activities of upstream signaling pathways. TLR4 is the receptor for LPS and its downstream MyD88-dependent pathway activates NF-κB and MAPK pathways, which in turn are responsible for the expression of costimulatory molecules and inflammatory cytokines (44, 45). Therefore, we compared the activities of these two pathways in LPS-treated *Tmod1^+/+^* and *TOT/Tmod1^-/-^* imDCs using western blotting. We found that IκB, which retains p65 in the cytosol, started to degrade within 5 min of LPS treatment and was almost undetectable by 30–60 min in *Tmod1^+/+^* imDCs (**Figure 3A**). However, in *TOT/Tmod1^-/-^* imDCs, although it started to become degraded within 5 min, it was still present in large amounts (1.7-fold relative to *Tmod1^+/+^*) at 30–60 min, suggesting that the degradation of IκB was retarded (**Figures 3A, B**). Correspondingly, p65 phosphorylation increased in 5 min and reached the highest level at 30 min in both *Tmod1^+/+^* and *TOT/Tmod1^-/-^* imDCs, but the phosphorylation level was considerably lower in *TOT/Tmod1^-/-^* imDCs than in *Tmod1^+/+^* imDCs (80% less at 5 min, **Figures 3A, B**). Although LPS-induced phosphorylation kinetics of p38 MAPK was similar between *Tmod1^+/+^* and *TOT/Tmod1^-/-^* imDCs, its phosphorylation level in *TOT/Tmod1^-/-^* imDCs was considerably lower than that in *Tmod1^+/+^* imDCs (30% less at 5 min, **Figures 3A, B**). No difference was found in the phosphorylation levels of ERK1/2 MAPK between the two groups (**Figure S4**). Our results indicate that in the absence of Tmod1, LPS-induced phosphorylation of the key signaling molecules, NF-κB and p38 MAPK, is significantly compromised.

Next, we examined the mRNA expression of costimulatory molecules and inflammatory cytokines. The data showed that *Tmod1^+/+^* and *TOT/Tmod1^-/-^* imDCs had low and comparable expression levels of *MHC-II*, *CD80*, *CD40*, and *CCR7*. However, the expression of CD86 was significantly lower in *TOT/Tmod1^-/-^* imDCs compared to *Tmod1^+/+^* imDCs (p < 0.001, **Figure 3C**). LPS treatment induced the upregulation of all the maturation markers in the two genotypes. The expression levels of *CD80* and *CD86* were significantly lower in *TOT/Tmod1^-/-^* mDCs than in *Tmod1^+/+^* mDCs (p < 0.05, **Figure 3C**), while expression levels of *MHC-II*, *CD40*, and *CCR7* were similar between the two groups. In terms of the mRNA expression of cytokines, we found that the expression of *Il6* and *Ifng* in *TOT/Tmod1^-/-^* mDCs was about 50% less than that in *Tmod1^+/+^* mDCs (p < 0.05, **Figure 3D**), but the expression of *Tnfa* and *Il12* was not significantly different between the two groups. These data suggest that Tmod1 deficiency has different and complex effects on the expression of costimulatory molecules and inflammatory cytokines.

### Tmod1 Deficiency Promotes the Activation of TLR4-TRIF-Dependent Pathway and Expression of Type I Interferons in DCs

**Figure 4:**
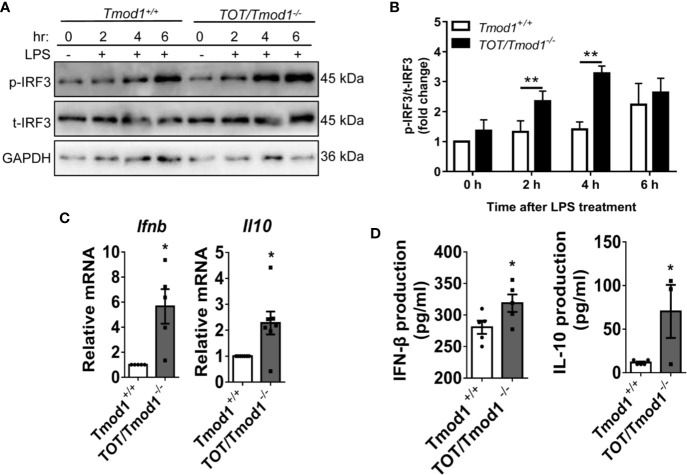
Tmod1 deficiency promotes the activation of TRIF-dependent pathway and expression of type I interferons in dendritic cells (DCs). **(A)** The immature DC (imDCs) from *Tmod1^+/+^* and *TOT/Tmod1^-/-^* mice were treated with LPS (100 ng/ml) for 0, 2, 4, 6 h, respectively, and then total proteins were extracted. The expression of phosphorylated IRF3 (p-IRF3) and total IRF3 (t-IRF3) were detected by western blotting. GAPDH were used as an internal control. **(B)** The expressions of p-IRF3 at different time points were quantified after normalization to t-IRF3 and the data was presented as fold change. **(C)** The imDCs from *Tmod1^+/+^* and *TOT/Tmod1^-/-^* mice were matured by LPS treatment for 2 days. Total RNAs were extracted from imDCs and mature DCs (mDCs) of both genotypes and qPCR was performed to detect the expression of IFN-β (*Ifnb*) and IL-10 (*Il10*). **(D)** Culture media were collected from lipopolysaccharide (LPS)-induced mDCs from *Tmod1^+/+^* and *TOT/Tmod1^-/-^* mice. The concentrations of IFN-β and IL-10 were measured on a flow cytometer by using a bead-based immunoassay kit. Data are presented as mean ± SEM. *p < 0.05, **p < 0.01, paired, two-tailed student’s *t*-test.

In addition to the MyD88-dependent pathway, the binding of LPS with TLR4 also triggers the activation of the TRIF-dependent pathway, resulting in the phosphorylation of IRF3 and the expression of type I interferons (46, 47). We treated imDCs of the two genotypes with LPS for 2, 4, and 6 h, and analyzed the phosphorylation of IRF3 using western blotting. The data showed that, following LPS treatment, IRF3 phosphorylation gradually increased and reached the highest level by 6 h in *Tmod1^+/+^* DCs. However, in *TOT/Tmod1^-/-^* DCs, the phosphorylation level of IRF3 was significantly higher than that in *Tmod1^+/+^* DCs at 2 and 4 h (p < 0.01) and remained high up to 6 h (**Figures 4A, B**). *Ifnb* and *Il10* are the two major downstream genes of IRF3 (9). We performed qPCR to compare their expression levels in LPS-induced *Tmod1^+/+^* and *TOT/Tmod1^-/-^* mDCs. We found that the expression of *Ifnb* and *Il10* was about 6- and 2- fold higher, respectively, in *TOT/Tmod1^-/-^* mDCs compared to that in *Tmod1^+/+^* mDCs (**Figure 4C**). Consistently, the concentrations of IFN-β and IL-10 secreted in the culture medium of *TOT/Tmod1^-/-^* mDCs were 318.8 ± 13.8 pg/ml and 70.4 ± 23.6 pg/ml, respectively, which were markedly higher than those of *Tmod1^+/+^* mDCs (280.6 ± 10.5 pg/ml and 11.7 ± 1.2 pg/ml, respectively) (**Figure 4D**). These data suggest that Tmod1 deficiency promotes the activation of the TRIF-dependent pathway and the expression of type I interferons.

Besides TLR4, other TLRs also signal *via* MyD88 or IRF or both, such as TLR2 agonist Pam3csk4 (MyD88-NF-κB pathway), TLR3 agonist poly (I:C) (TRIF-IRF3 pathway), and TLR9 agonist CpG ODN 1826 (MyD88-NF-κB and -IRF7 pathway) (48). To examine whether Tmod1 was required for signaling through other TLRs, we stimulated *Tmod1^+/+^* and *TOT/Tmod1^-/-^* imDCs with LPS, Pam3csk4, poly (I:C), and CpG. We observed a significant decrease in *Il6* mRNA level and markedly augmentation in *Il10* and *Ifnb* mRNA levels by LPS in *TOT/Tmod1^-/-^* imDCs (**Figure S5A, C, D**). But Pam3csk4, poly (I:C), and CpG failed to induce changes in *Il6*, *Tnfa*, *Il10*, and *Ifnb* mRNA expression in *TOT/Tmod1^-/-^* imDCs (**Figure S5A–D**). Consistently, TNF-α secretion was significantly inhibited and IL-10 secretion was considerably enhanced by LPS in *TOT/Tmod1^-/-^* imDCs, while other TLR agonists did not show much effect (**Figure S5E, F**). These data suggest that Tmod1 may have a unique function on TLR4 signaling.

### Exogenous Tmod1 Expression Reversed the Effect of Tmod1 Deficiency on TLR4 Signaling

**Figure 5:**
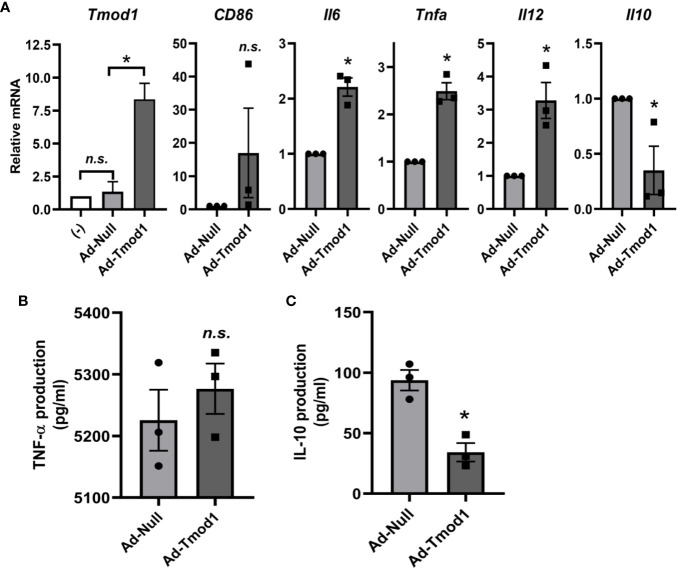
Exogenous Tmod1 expression reversed the effect of Tmod1 deficiency on Toll-like receptor 4 (TLR4) signaling. **(A)** Immature dendritic cells (ImDCs) from *TOT/Tmod1^-/-^* mice were infected with Tmod1 adenovirus (Ad-Tmod1) or control adenovirus (Ad-Null) at 200 multiplicity of infection (MOI) for 6 h. ImDCs without adenovirus treatment were used as the negative control (-). Adenovirus infected or un-infected imDCs were further treated with LPS (100 ng/ml) for 24 h and total RNAs were extracted. The mRNA expression of *Tmod1*, MyD88 downstream genes (*CD86*, *Il6*, *Tnfa*, and *Il12*) and TRIF/IRF3 downstream gene (*Il10*) were detected by qPCR. **(B, C)** Culture media were collected at the end of lipopolysaccharide (LPS) treatment. The concentrations of cytokines, TNF-α **(B)** and IL-10 **(C)**, were measured by ELISA kits. Data are presented as mean ± SEM. *p < 0.05, *n.s.*, no statistical significance; paired, two-tailed student’s *t*-test.

Since Tmod1 deficiency resulted in inhibited TLR4/MyD88 signaling and enhanced TLR4/TRIF/IRF3 signaling, we next sought to explore whether exogenous Tmod1 could rescue these effects. We introduced exogenous Tmod1 into *TOT/Tmod1^-/-^* imDCs by adenovirus infection and then treated cells with LPS. The expressions of MyD88 and TRIF/IRF3 downstream genes were measured by qPCR and/or ELISA. qPCR data confirmed the re-expression of Tmod1 in Ad-Tmod1 infected cells (**Figure 5A**, left panel). Compared with Ad-Null infected mDCs, the expression of maturation marker, *CD86*, was increased to some extent in Ad-Tmod1 infected mDCs (**Figure 5A**, second panel). Strikingly, the expressions of *Il6*, *Tnfa*, and *Il12* were all significantly elevated, while the expression of *Il10* was greatly reduced (p < 0.05, **Figure 5A**, right panels). Furthermore, ELISA data showed that TNF-α secretion in Ad-Tmod1 infected mDCs was increased a little bit but did not show significance (**Figure 5B**). Yet, IL-10 secretion was significantly decreased (p < 0.05, **Figure 5C**). These data suggest that rescue with exogenous Tmod1 expression in Tmod1-deficient DCs made MyD88-dependent pathway more activated and TRIF-dependent pathway more inhibited, thus, it reversed the effect of Tmod1 deficiency on TLR4 signaling.

### Tmod1 Deficiency Did Not Affect the Endocytosis but Impaired the Migration Ability of DCs

**Figure 6:**
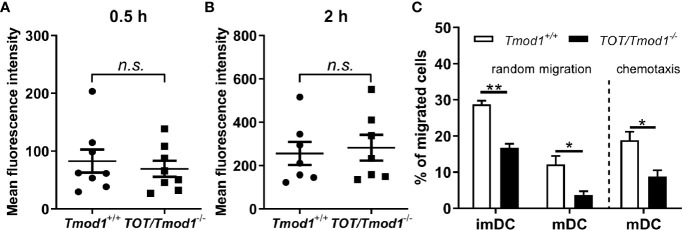
Tmod1 deficiency did not affect the endocytosis but impaired the migration ability of dendritic cells (DCs). **(A, B)** Immature DCs (ImDCs) from *Tmod1^+/+^* and *TOT/Tmod1^-/-^* mice were incubated with FITC-labeled dextran for 0.5 h **(A)** and 2 h **(B)**, and their fluorescence intensities were analyzed by flow cytometry. **(C)** The imDCs and lipopolysaccharide (LPS)-induced mature DCs (mDCs) from *Tmod1^+/+^* and *TOT/Tmod1^-/-^* mice were plated in the upper compartment of the Boyden chamber and the culture medium for imDCs or mDCs was added to the lower compartment. After incubation for 12 h, the numbers of randomly migrated cells were counted from the lower compartment. To measure the chemotaxis of mDCs, culture medium supplemented with CCL19 was placed in the lower compartment of Boyden chamber. The numbers of migrated mDCs were counted after 12 h. Data are presented as mean ± SEM. *p < 0.05, **p < 0.01, *n.s.*, no statistic significance; unpaired, two-tailed student’s *t*-test.

Endocytosis of antigens, migration, and antigen presenting abilities are the most important immune functions of DCs. Therefore, we investigated whether Tmod1 deficiency affects these immune functions. ImDCs, residing in the peripheral tissues, engulf pathogens and antigens by endocytosis. We incubated *Tmod1^+/+^* and *TOT/Tmod1^-/-^* imDCs with FITC-labeled dextran for 0.5 and 2 h and detected their fluorescence intensities using flow cytometry. The data showed that the mean fluorescence intensities at 0.5 h were comparable between the two genotypes (82.7 ± 20.0 vs. 69.5 ± 13.5, **Figure 6A**). The mean fluorescence intensities increased considerably at 2 h, suggesting that more endocytosis occurred, but there was no difference between the two groups (**Figure 6B**). This indicates that Tmod1 deficiency did not affect the endocytosis ability of imDCs.

ImDCs patrol in the periphery by random migration, while mDCs undergo directional migration to lymph nodes because of high CCR7 expression (49, 50). Therefore, we used a modified Boyden chamber to measure the random migration of imDCs and mDCs, and chemotactic migration of mDCs in the two genotypes. We found that *TOT/Tmod1^-/-^* imDCs and mDCs showed significantly slower random migration compared to their *Tmod1^+/+^* counterparts (p < 0.01 and p < 0.05, respectively, **Figure 6C**, left panel). When we measured the chemotaxis of mDCs toward CCL19, we found that the percentage of migrated *TOT/Tmod1^-/-^* mDCs was half of that of migrated *Tmod1^+/+^* mDCs (**Figure 6C**, right panel). These data suggest that knockout of Tmod1 impaired the migration abilities of both imDCs and mDCs.

### Tmod1 Deficiency Impaired the T-Cell Stimulatory Ability of Mature DCs and Induced Immune Tolerance in the EAE Model

**Figure 7:**
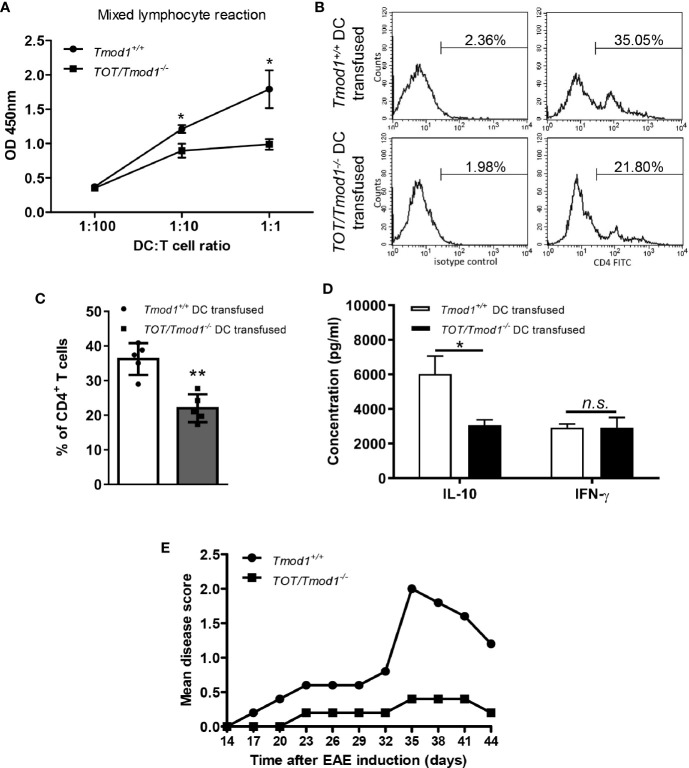
Tmod1 deficiency impaired the T-cell stimulatory ability of mature dendritic cells (mDCs) and induced immune tolerance in the experimental autoimmune encephalomyelitis (EAE) model. **(A)** LPS-induced mDCs from *Tmod1^+/+^* and *TOT/Tmod1^-/-^* mice were incubated with 1×10^5^ T cells in 1:100, 1:10, 1:1 ratios in the mixed lymphocytes reaction. The proliferation of T cells was measured by cell counting kit-8 and optical densities at 450 nm (OD450) were obtained. **(B, C)** Lipopolysaccharide (LPS)-induced mDCs from *Tmod1^+/+^* and *TOT/Tmod1^-/-^* mice (N=5) were treated with OVA peptide (323-339) and then transfused, respectively, to C57BL/6J mice at day 1, 3, and 5. On day 8, the spleen cells of transfused mice were collected and stimulated with OVA peptide (323-339) for another 3 days. The stimulated spleen cells were stained with FITC-conjugated anti-CD4 antibody or isotype control and analyzed by flow cytometry. The representative histograms were presented **(B)** and the percentages of CD4^+^ T cells were obtained **(C)**. **(D)** The culture media of OVA peptide-stimulated spleen cells were collected and the concentrations of IL-10 and IFN-γ were measured on a flow cytometer by using a bead-based immunoassay kit. **(E)** LPS-induced mDCs from *Tmod1^+/+^* and *TOT/Tmod1^-/-^* mice (N=5) were pulsed with MOG35-55 peptide and transfused, respectively, to C57BL/6J mice on day 0 and day 4. EAE was induced by injection of MOG35-55 peptide and *Mycobacterium tuberculosis* on day 7 and 14, and pertussis toxin on day 7 and 9. The mice were observed and scored from day 14 until day 44. The mean disease scores were plotted. Data are presented as mean ± SEM. *p < 0.05. **p < 0.01, *n.s.*, no statistical significance; unpaired, two-tailed student’s *t*-test.

mDCs are mainly responsible for presenting antigens to naïve T cells and priming them to produce inflammatory cytokines such as IFN-γ and IL-10, and then mediating immune responses (15). Therefore, we sought to determine whether Tmod1 deficiency affects the T-cell stimulatory abilities of mDCs *in vitro* and *in vivo*. Allogeneic MLR data showed that *Tmod1^+/+^* mDCs substantially promoted the proliferation of T cells, while the proliferation of T cells mixed with *TOT/Tmod1^-/-^* mDCs was significantly reduced (p < 0.05, **Figure 7A**). To further validate this data, an *in vivo* assay was performed (41), in which C57BL/6J mice were transfused with OVA peptide-loaded *Tmod1^+/+^* and *TOT/Tmod1^-/-^* mDCs, respectively, and their spleen cells were collected and re-stimulated with OVA peptide for 3 days. The spleen cells were stained with FITC-conjugated CD4 antibody and subjected to flow cytometry analysis. We found that the percentage of CD4^+^ T cells in the spleen cells from *TOT/Tmod1^-/-^* mDC transfused mice was 22.02 ± 1.80%, which was significantly lower than that in spleen cells from *Tmod1^+/+^* mDC transfused mice (36.23 ± 2.05%) (p < 0.01, **Figures 7B, C**). We also measured the concentrations of IFN-γ and IL-10 in the culture medium of the spleen cells by flow cytometry. The data showed that the spleen cells from *TOT/Tmod1^-/-^* mDC transfused mice produced markedly less IL-10 compared to those from *Tmod1^+/+^* mDC transfused mice (3062.2 ± 311.1 pg/ml vs. 6021.7 ± 1032.7 pg/ml, p < 0.05) (**Figure 7D**). However, the production of IFN-γ was similar between the two groups (**Figure 7D**). These data suggest that *TOT/Tmod1^-/-^* mDCs were defective in T cell activation and priming. Moreover, Tmod1-deficient mDCs impaired the production of IL-10-producing T cells, but not IFN-γ-producing T cells. The results also indicate that Tmod1-deficient mDCs may be able to induce peripheral tolerance.

To test this hypothesis, we compared the abilities of *Tmod1^+/+^* and *TOT/Tmod1^-/-^* mDCs to prevent the development of EAE in mice, which was induced through subcutaneous injection of an encephalitogenic MOG peptide. The data showed that the mean disease score in mice injected with *Tmod1^+/+^* mDCs increased gradually from day 14 to day 35 (0 to 2.0), and then dropped after day 35. However, the mean disease score in mice injected with *TOT/Tmod1^-/-^* mDCs remained < 0.5 from day 14 to day 44 (**Figure 7E**), indicating that three prior injections of MOG peptide-pulsed *TOT/Tmod1^-/-^* mDCs offered almost completely protection against the neuropathological symptoms associated with EAE. This suggests that Tmod1-deficient mDCs induced tolerance to the MOG peptides.

### Tmod1 Deficiency Diminished LPS-Induced F-Actin Formation and Cell Stiffening in DCs

**Figure 8:**
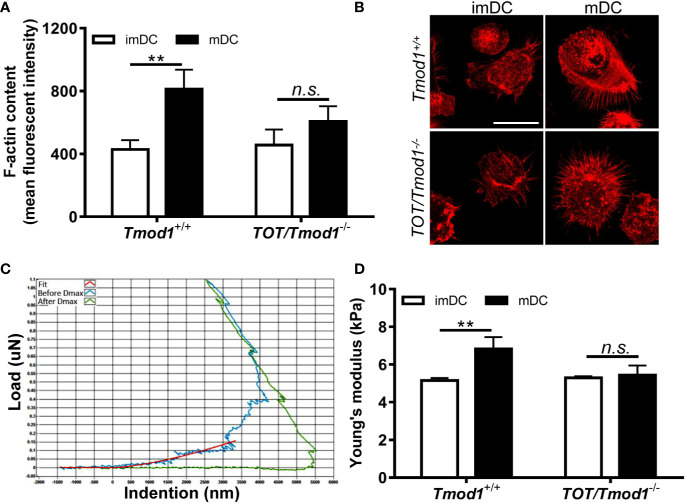
Tmod1 deficiency diminished lipopolysaccharide (LPS) induced F-actin formation and cell stiffening in dendritic cells (DCs). **(A)** The immature DCs (imDCs) from *Tmod1^+/+^* and *TOT/Tmod1^-/-^* mice were treated with LPS for 2 days to induce maturation. imDCs and mature DCs (mDCs) of both genotypes were stained with rhodamine phalloidin and subjected to flow cytometry analysis. The mean fluorescence intensities were obtained. **(B)** The imDCs and mDCs from *Tmod1^+/+^* and *TOT/Tmod1^-/-^* mice were cultured on poly L-Lysine-treated coverslips and stained with rhodamine phalloidin. The fluorescent signals were observed on the confocal microscopy. Representative images were shown. Bar=25 μm. **(C, D)** The imDCs and mDCs of *Tmod1^+/+^* and *TOT/Tmod1^-/-^* mice were placed on a nanoindenter to measure the cell stiffness. The representative indention-load curve was shown **(C)**. The curves were fitted using the Hertzian contact model and the Young’s moduli of cells (N=20~25) were calculated **(D)**. Data are presented as mean ± SEM. **p < 0.01, *n.s.*, no statistical significance; unpaired, two-tailed student’s *t*-test.

The immune functions of DCs rely on their cytoskeletal structure and biomechanical properties. It has been shown that F-actin is increased in LPS-stimulated DCs (21). Since Tmod1 regulates the polymerization of actin filaments, it may participate in LPS-induced F-actin remodeling. Therefore, we analyzed the F-actin content in imDCs and mDCs of *Tmod1^+/+^* and *TOT/Tmod1^-/-^* mice using flow cytometry and fluorescence microscopy. As shown in **Figures 8A, B**, the F-actin content was significantly increased in *Tmod1^+/+^* mDCs compared to *Tmod1^+/+^* imDCs (p < 0.01). However, no difference was found in the F-actin contents of *TOT/Tmod1^-/-^* imDCs and mDCs. We further measured the cell stiffness of imDCs and mDCs using a nanoindenter, in which the cells were indented with a probe and the loading (blue) and unloading (green) curves were drawn (**Figure 8C**). Young’s modulus was calculated by fitting the curves with the Hertzian contact model. We found that *Tmod1^+/+^* mDCs had greater Young’s moduli than *Tmod1^+/+^* imDCs, while *TOT/Tmod1^-/-^* imDCs and mDCs had similar Young’s moduli (**Figure 8D**). Our data suggest that Tmod1 deficiency diminished LPS-induced F-actin formation and cell stiffening in the DCs.

## Discussion

In the present study, we show evidence that Tmod1, an actin-capping protein, is expressed in DCs and plays indispensable roles in controlling the maturation and multiple immune functions of DCs. Although Tmod1 was not essential for the differentiation of DCs, it was differentially expressed and had distinct functions in DC maturation. Tmod1 expression level was relatively low in imDCs (**Figure 1**). Tmod1 deficiency did not affect their endocytosis, but substantially impaired their free migration abilities (**Figure 6**), suggesting that Tmod1 has limited roles in the immune functions of imDCs. Tmod1 was upregulated in LPS-induced mDCs, which may be a result of the activation of the NF-κB signaling pathway (51). Importantly, our data showed that Tmod1 regulates the expression of costimulatory molecules and chemokine receptors (**Figure 2**), secretion of cytokines (**Figure 2**), migration abilities (**Figure 6**), and T-cell stimulatory abilities of mDCs (**Figure 7**).

The upregulation of MHC-II, costimulatory molecules, and chemokine receptors, and the secretion of inflammatory cytokines are the key features of DC maturation (7). The significantly low cell surface expression of MHC-II, CD80, CD86, CD40, and CCR7, and the decreased secretion of TNF-α, IL-12(p70), and IFN-γ in *TOT/Tmod1^-/-^* mDCs suggests that *TOT/Tmod1^-/-^* mDCs are less mature than *Tmod1^+/+^* mDCs (**Figure 2**). This indicates that Tmod1 plays a critical role in regulating LPS-induced DC maturation. The underlying mechanisms may lie in the effects of Tmod1 on the TLR4-mediated signaling pathways. Downstream of TLR4, the MyD88-dependent NF-κB and MAPK pathways control the expression of costimulatory molecules and proinflammatory cytokines (52–55). Western blotting data showed that compared to *Tmod1^+/+^* DCs, degradation of IκB was delayed and phosphorylation of p65 and p38 was reduced in *TOT/Tmod1^-/-^* DCs under LPS stimulation (**Figures 3A, B**). This suggests that the NF-κB and p38 MAPK pathways were significantly inhibited when Tmod1 was deficient. Consistently, the gene expression levels of CD80, CD86, IL-6, and IFN-γ were found to be decreased in LPS-treated *TOT/Tmod1^-/-^* DCs (**Figures 3C, D**). We noticed that although the gene expression of MHC-II, CD40, CCR7, IL12, and TNF-α did not change in the LPS-treated *TOT/Tmod1^-/-^* DCs, their surface expression or secretion were decreased (**Figure 2**). It has been shown that MHC-II is translocated onto the membrane when DCs mature (56). The secretion of inflammatory cytokines involves the trafficking of endosomal compartment (57). There is mounting evidence showing that the cytoskeleton can provide a scaffold for intracellular material transportation (58, 59). Therefore, Tmod1 may affect the trafficking of these membrane molecules and cytokines by regulating the actin cytoskeleton, rather than regulating their mRNA expression. This also indicates that Tmod1 has complex effects on the DC maturation process.

The TRIF/IRF3 pathway, activated when TLR4 is endocytosed into the endosomal compartments, promotes the expression of anti-inflammatory cytokines (9, 60, 61). The induction of IRF3 does not occur from plasma membrane-located TLR4 (62). Our data showed that after LPS treatment, phosphorylation of IRF3 was considerably higher in *TOT/Tmod1^-/-^* DCs than in *Tmod1^+/+^* DCs (**Figures 4A, B**). The expression and secretion of anti-inflammatory cytokines, IFN-β and IL-10, were also found to be significantly increased in *TOT/Tmod1^-/-^* DCs (**Figures 4C, D**). These results suggest that activation of the TRIF/IRF3 pathway was enhanced, and thus the endocytosis of TLR4 might be augmented when Tmod1 was deficient. It was shown that the release of IL-10, during DC maturation, could interfere with the upregulation of costimulatory molecules, CD80/86, and the production of proinflammatory cytokines, such as INF-γ, TNF-α, and IL-12 (63–67). This autocrine effect of IL-10 may be a reason for *TOT/Tmod1^-/-^* mDCs to display an immature phenotype. In addition, in TLR4 signaling, MyD88-dependent proinflammatory and TRIF-dependent anti-inflammatory pathways are competitive and thus restrict the activation of one another (68). Therefore, the enhanced activation of the TRIF/IRF3 pathway in Tmod1-deficient DCs may negatively regulate the MyD88-dependent pathway. The question remains as to how Tmod1 regulates TLR4 endocytosis and TRIF/IRF3 pathway. Our preliminary data showed that Tmod1 deficiency did not affect the expression of TLR4 and MyD88 in DCs, but significantly upregulated the expression of CD14, which controls LPS-induced endocytosis of TLR4 (69, 70). Consistently, in macrophages overexpressing Tmod1, we observed the downregulated CD14 expression and less localization of TLR4 in early endosome and lysosome. However, further studies are needed to understand the precise mechanism through which Tmod1 regulates the expression of CD14.

It should be pointed out that the alteration of TLR4 signaling induced by Tmod1 deficiency did not come from the compensatory effect of other Tmod homologs (**Figure S2**). Moreover, rescue with exogenous Tmod1 expression in *TOT/Tmod1^-/-^* DCs reversed the effect of Tmod1 deficiency on TLR4 signaling (**Figure 5**), providing a strong support for the important role of Tmod1 in regulating TLR4 signaling. Another interesting finding is that Tmod1 deficiency seemed to have no obvious effect on other TLR signaling (**Figure S3**), such as TLR2, TLR3, and TLR9, which also signals *via* MyD88 or TRIF/IRF (48). Considering that only LPS but not other TLRs could induce upregulation of Tmod1 in DCs (**Figure S1**), we speculate that Tmod1 may have a unique function in TLR4 signaling, which needs to be further explored.

Upon maturation, DCs gain the ability of chemotactic migration, which enables them to migrate from the sites of infection to the secondary lymphoid organs (14). This process is coordinated by the CCR7 ligands, CCL19 and CCL21 (71). Our data showed that compared with *Tmod1^+/+^* mDCs, *TOT/Tmod1^-/-^* mDCs displayed impaired chemotaxis to CCL19 (**Figure 6C**). This could be well explained by the reduced expression level of CCR7 in *TOT/Tmod1^-/-^* mDCs (**Figure 2**). Further, like in imDCs, Tmod1 deficiency also resulted in impaired ability of random migration in the mDCs. This indicates that Tmod1 plays an important role in regulating both random migration and chemotaxis throughout the lifetime of DCs. It is known that DCs adopt the mesenchymal migration mode to move in the extracellular matrix, which includes three classic steps: spreading of the leading edge, adhesion to substrates, and retraction of the uropods (72–74). A simulation of the random and directed motion of DCs showed that in a strong chemotactic field, the DCs orient their filopodia to one pole, while in random migration, the filopodia are organized randomly (75). The places where filopodia appear are determined by spatially organized actin polymerization centers around the cell periphery. Tmod1 may participate in the filopodia formation by regulating actin polymerization centers. Indeed, in Tmod1-deficient mDCs, actin polymerization (i.e., F-actin formation) and cell stiffening induced by LPS were abolished (**Figure 8**). Retraction of the uropod is mediated by stress fibers, which produce traction force promoting the cell to move forward (74). A recent study showed that Tmod1 is the key components of contractile stress fibers in non-muscle cells (30). Therefore, Tmod1 may also affect the stress fibers and uropod retraction during DC migration.

The mission of mDCs is to present an antigen peptide to naïve T cells to subsequently initiate immune responses (14). Full activation of T cells requires two signals, i.e., the antigens held in MHC complex and costimulatory molecules on DCs, and the effect of cytokines (76, 77). T-cell activation is the basis for its further proliferation and expansion. Both allogeneic MLR and *in vivo* assays showed that *TOT/Tmod1^-/-^* mDCs had impaired ability to promote the proliferation of CD4^+^ T cells (**Figure 7**), suggesting that they were incompetent in stimulating T cells. This defect should result from the low expression of MHC-II and costimulatory molecules, CD80/86 and CD40, on the membrane of *TOT/Tmod1^-/-^* mDCs (**Figures 2A, B**). The reduced secretion of cytokines, such as TNF-α, IFN-γ, and IL-12, in *TOT/Tmod1^-/-^* mDCs (**Figure 2C**) would further worsen this defect. The formation of immunological synapse between T cells and DCs involves rearrangement of the F-actin in the cell membrane into polymers (78–80). The reduction of F-actin content in *TOT/Tmod1^-/-^* mDCs (**Figures 8A, B**) may make it difficult to form immune synapses with T cells. Moreover, the impaired chemotactic migration would also prevent *TOT/Tmod1^-/-^* mDCs from migrating to lymphoid tissues and thus decrease the possibility of DC and T-cell interaction *in vivo*. Therefore, Tmod1 deficiency in mDCs resulted in defective antigen presentation, inadequate T-cell activation, and thus immune tolerance. This is well supported by the fact that *TOT/Tmod1^-/-^* mDCs almost completely prevented the occurrence of neuropathological symptoms in the EAE model (**Figure 7E**).

Recent studies have shown that in addition to imDCs and mDCs, there is an intermediate population of DC maturation, called semi-mature DCs or tolerogenic DCs (DCreg) (81, 82). The main characteristics of this population are: 1) low or normal expression of cell surface markers, including MHC-II and costimulatory molecules; 2) increased secretion of anti-inflammatory cytokines (such as IL-10, retinoic acid, and TGF-β); 3) decreased production of proinflammatory cytokines (such as IL-12, IL-6, TNF-α, and IFN-γ); and 4) mediating immune tolerance (83, 84). Careful observation revealed that LPS-induced *TOT/Tmod1^-/-^* mDCs possessed almost all the characteristics of semi-mature DCs. This indicates that Tmod1 is an important molecule necessary for DC maturation.

In conclusion, our study identified the important roles of Tmod1 in DC maturation and multiple aspects of the immune functions of DCs. Tmod1 may be a potential target for regulating DC functions in order to exaggerate or suppress the immune responses in the body, which would be beneficial to the immunotherapy for many diseases including cancer, transplant rejection, infection, and autoimmune diseases.

## Data Availability Statement

The original contributions presented in the study are included in the article/**Supplementary Material**. Further inquiries can be directed to the corresponding authors.

## Ethics Statement

The animal study was reviewed and approved by The Ethics committee of Peking University Health Science Center.

## Author Contributions

XL and XX conducted the experiments, analyzed the results, and wrote the paper. XW, JZ, JL, and XG assisted with various experiments. LAS provided the knockout and rescued mouse lines and reviewed the paper. WY and ZZ conceptualized and supervised the project, analyzed the data, and wrote the paper. All authors contributed to the article and approved the submitted version.

## Funding

This work was funded by grants from the National Natural Science Foundation of China (31570938, 31771280, 31771014, 31660258, 11762006), the Science and Technology Innovative Talent Team of Guizhou Province (2015-4021), the High-level Innovative Talents Training Program of Guizhou Province - “100” Level Talents (2016-5676), the Science and Technology Foundation of Guizhou Province (2018-1412), and the Natural Science Research Project of Education Department of Guizhou Province (YJSCXJH 2019-068).

## Conflict of Interest

The authors declare that the research was conducted in the absence of any commercial or financial relationships that could be construed as a potential conflict of interest.
