# Supplementary material for: Tropomodulin1 Expression Increases Upon Maturation in Dendritic Cells and Promotes Their Maturation and Immune Functions
Source: Front Immunol. 2021 Jan 15;11:587441. doi: 10.3389/fimmu.2020.587441 (PMC7856346; doi:10.3389/fimmu.2020.587441)
Supplement: Supplementary file 1 [file DataSheet_1.docx]

**Supplementary Data**


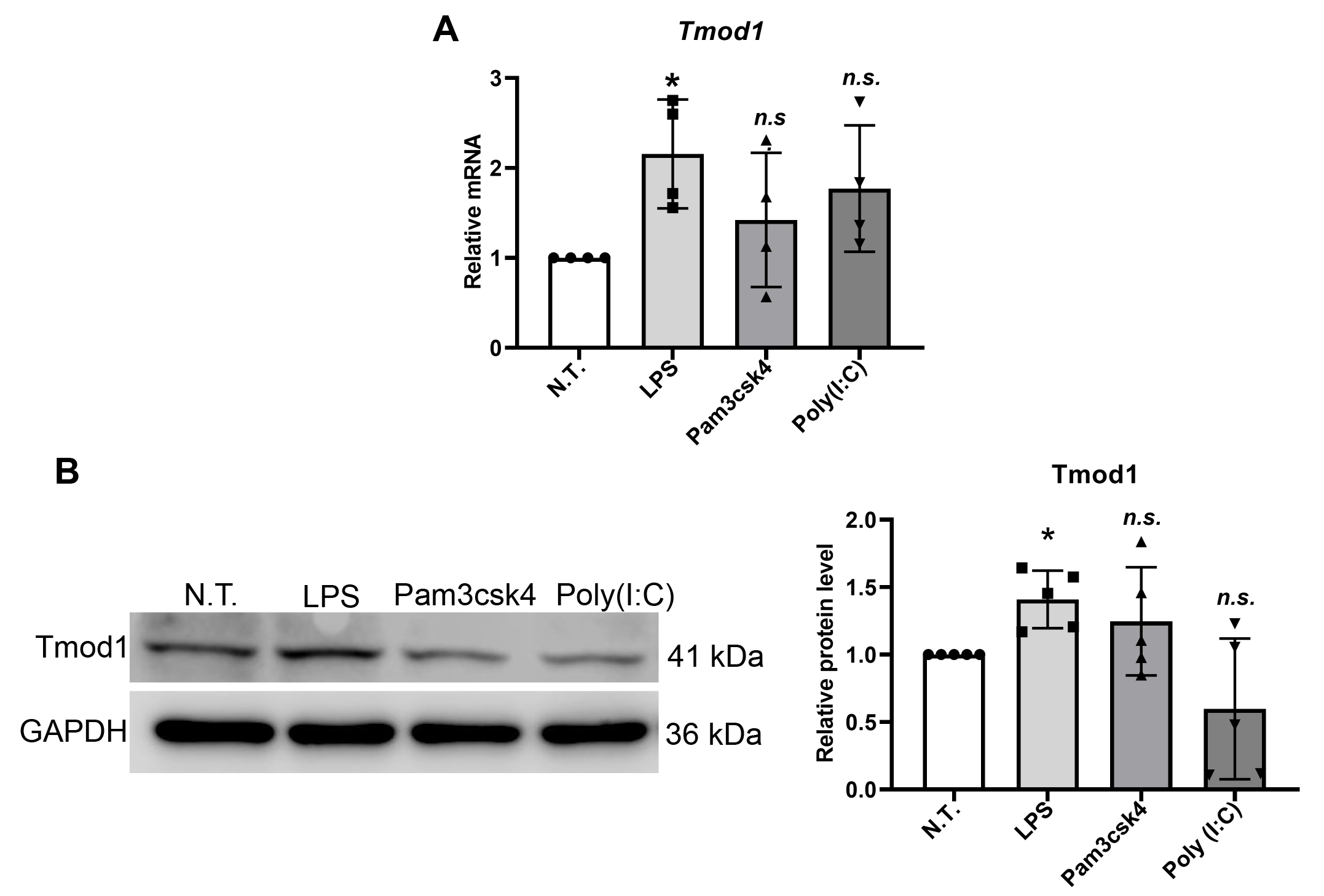


**Figure S1.** Tmod1 expression in DCs stimulated with TLR agonists. Immature DCs of wild type mice were stimulated with LPS (for TLR4, 100 ng/mL), Pam3csk4 (for TLR2, 100 ng/mL), or poly(I:C) (for TLR3, 25 μg/mL) for 24 or 48 h. Total RNAs and proteins were isolated. **(A)** The mRNA level of *Tmod1* was measured by qPCR and fold changes were shown. N.T. stands for no treatment. **(B)** The protein level of Tmod1 was detected by western blotting. GAPDH was used for internal control. The bands were quantified by Image J software. Data are presented as mean ± SEM. As compared to N.T., *: p < 0.05, *n.s.*: no statistic significance; paired, two-tailed student’s *t*-test.

**
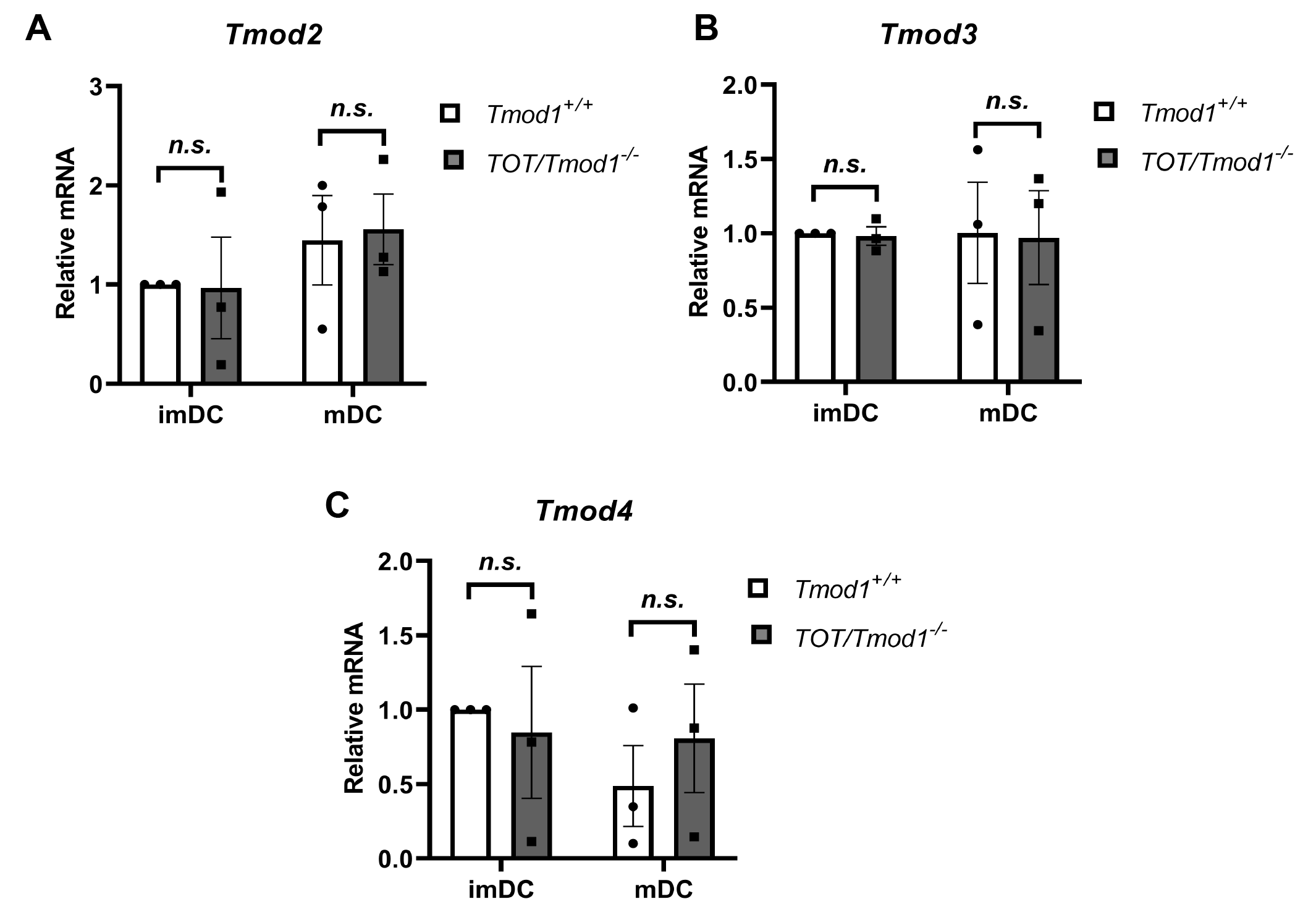
**

**Figure S2.** The gene expression of Tmod homologs in Tmod1-deficient DCs. The imDCs of *Tmod1^+/+^* and *TOT/Tmod1^-/-^* mice were treated with LPS (100 ng/mL) for 48 h to induce maturation. Total RNAs were extracted and qPCR was performed to detect the mRNA level of Tmod homologs, *Tmod2* (A), *Tmod3* (B), and *Tmod4* (C). Data are presented as mean ± SEM. *n.s.*: no statistic significance; paired, two-tailed student’s *t*-test.

**
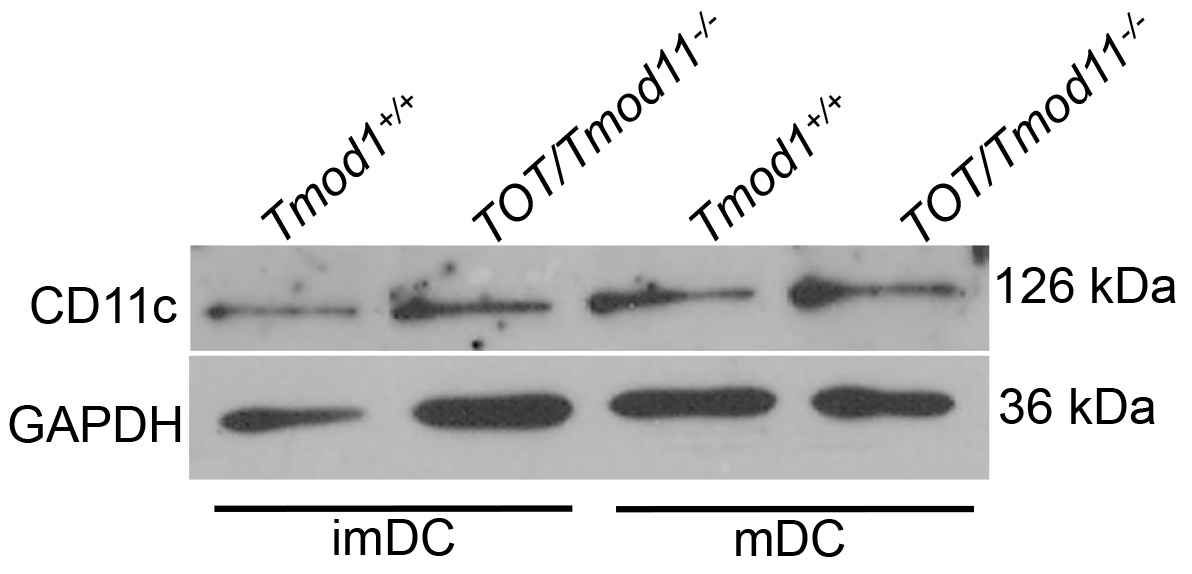
**

**Figure S3.** The expressions of CD11c in imDCs and mDCs of *Tmod1^+/+^* and *TOT/Tmod1^-/-^* mice as detected by western blotting. The imDCs of *Tmod1^+/+^* and *TOT/Tmod1^-/-^* mice were treated with LPS (100 ng/mL) for 48 h to induce maturation. Total proteins were isolated and western blotting was performed to detect the protein level of CD11c. GAPDH was used as an internal control. A representative blot was shown.


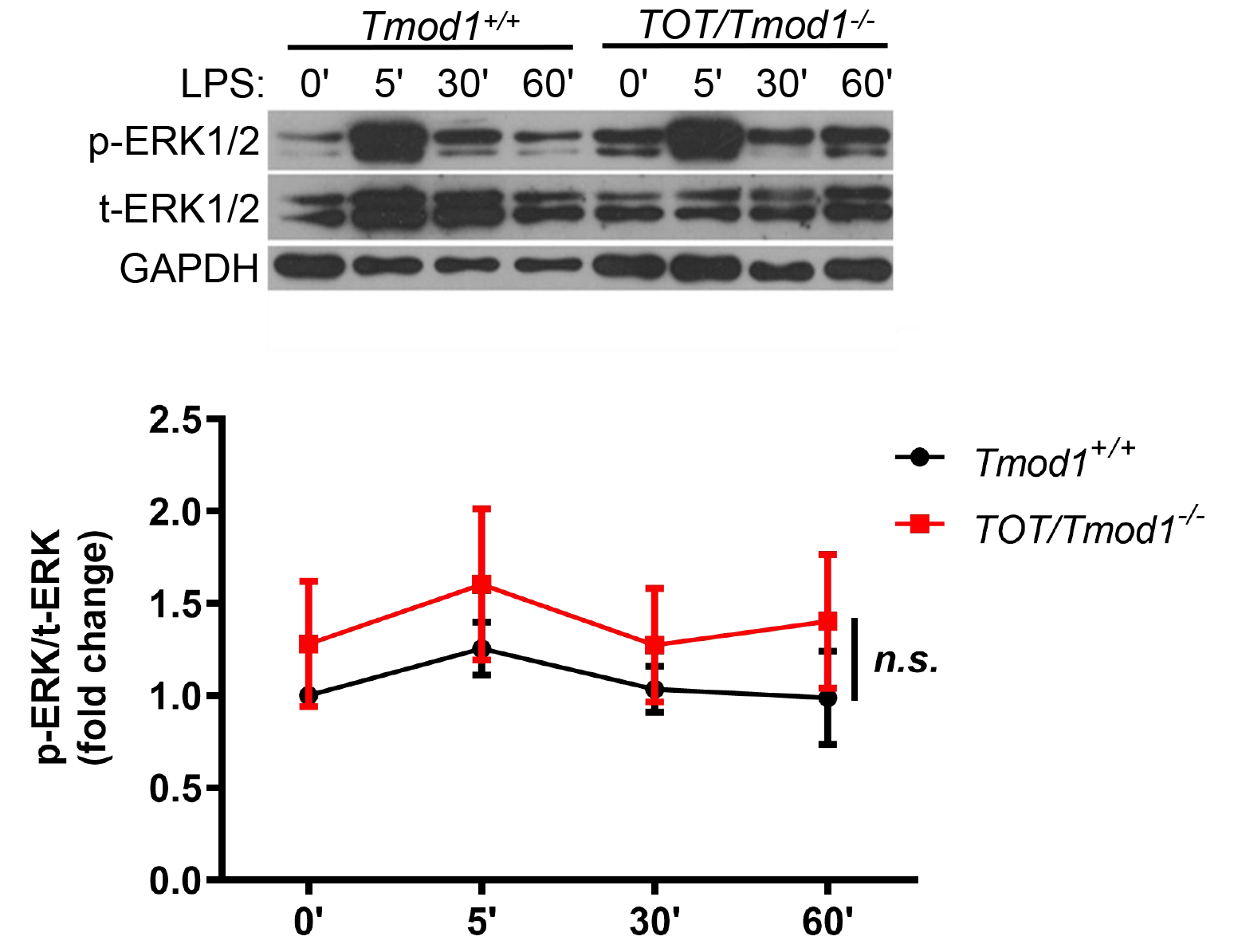


**Figure S4.** The activity of ERK1/2 signaling pathway in Tmod1-deficient DCs. The imDCs from *Tmod1^+/+^* and *TOT/Tmod1^-/-^* mice were treated with LPS (100 ng/ml) for 0, 5, 30 and 60 min, respectively, and then total proteins were isolated. The expression of phosphorylated ERK1/2 (p-ERK1/2) and total ERK1/2 (t-ERK1/2) were detected by western blotting. GAPDH were used as an internal control. A representative blot was shown (top panel). The expressions of p-ERK1/2 at different time points were quantified after normalization to t-ERK1/2 and fold changes were calculated (bottom panel). Data from five biological repeats are presented as mean ± SEM. *n.s.*: no statistical significance; 2-way ANOVA.

**
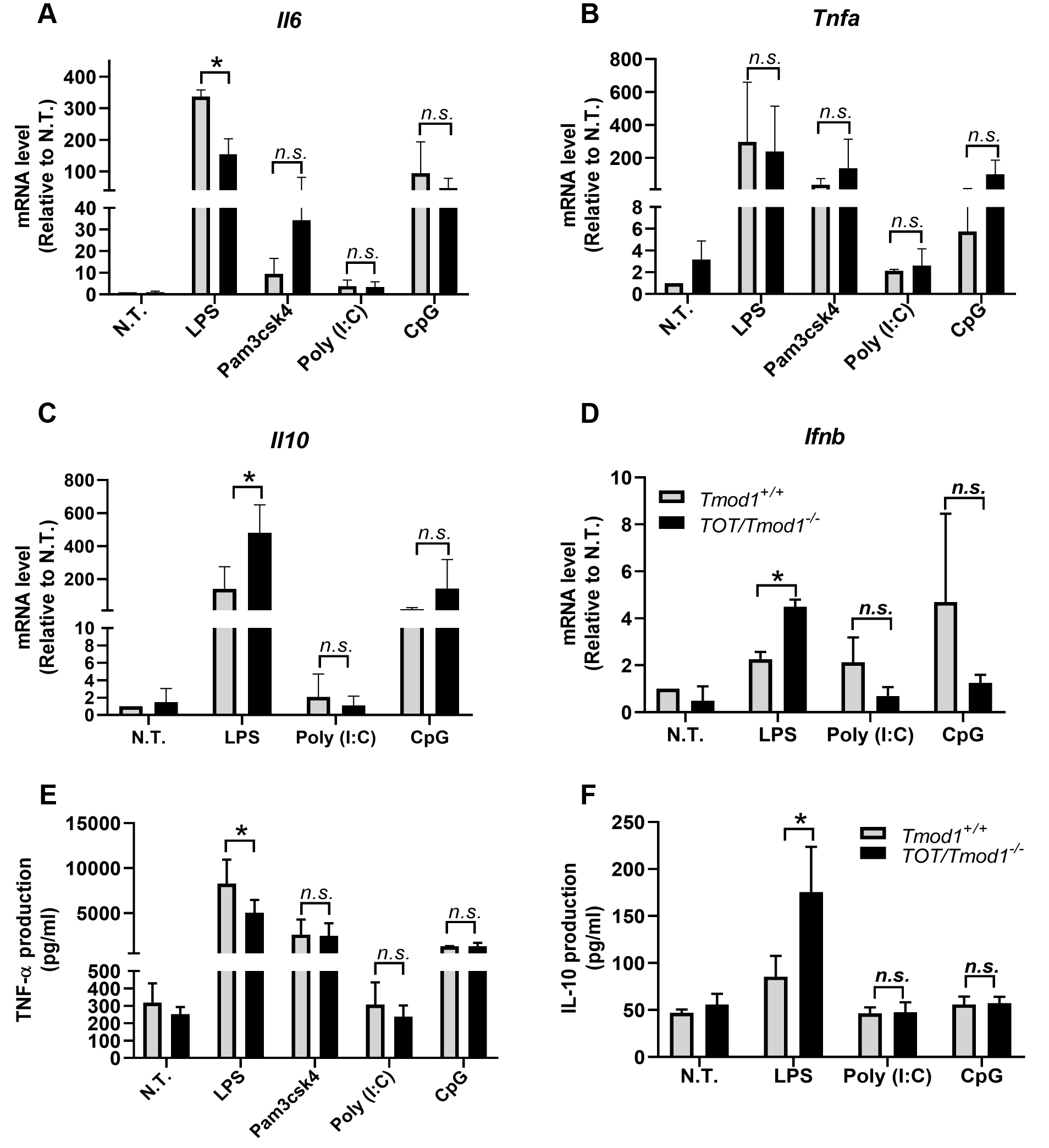
**

**Figure S5.** The gene expression and cytokine production in *Tmod1^+/+^* and *TOT/Tmod1^-/-^* imDCs after stimulation with TLR agonists. ImDCs of *Tmod1^+/+^* and *TOT/Tmod1^-/-^* mice were stimulated with LPS (100 ng/mL), Pam3csk4 (100 ng/mL), Poly (I:C) (25 μg/mL), or CpG ODN 1826 (an agonist for TLR9, 1μM) for 24 h. **(A-D)** Total RNAs were extracted and mRNA expression levels of *Il6* (A), *Tnfa* (B), *Il10* (C), and *Ifnb* (D) were detected by qPCR. **(E-F)** The culture media of imDCs stimulated with TLR agonists were collected and the concentrations of TNF-α (E) and IL-10 (F) were measured by ELISA kits. Data are presented as mean ± SEM. *: p < 0.05, *n.s.*: no statistic significance; paired, two-tailed student’s *t*-test.
